# Documentation of chaperone presence in breast clinic, a complete audit cycle

**DOI:** 10.1007/s11845-021-02634-y

**Published:** 2021-05-10

**Authors:** Aqeel Alameer, Amira Mohammed, Sami Abd Elwahab, Michael Boland, Amr Elfadul, Tej Tiwary, Colm Power, Michael Allen, Abeeda Butt, Arnold Hill

**Affiliations:** 1 Department of Surgery, Beaumont Hospital, Royal College of Surgeons, Dublin, Ireland; 2 Breast Clinic, Beaumont Hospital, Royal College of Surgeons, Dublin, Ireland

**Keywords:** Audit, Breast clinic, Chaperone, IMC guidelines

## Abstract

**Objective:**

The General Medical Council (GMC) and Irish Medical Council (IMC) recommend the presence of a chaperone for all intimate examinations and that it should be clearly documented. The aim of this report is to assess doctors’ compliance with obtaining a chaperone and documenting their presence, determining possible causes of non-compliance and implement interventions to increase compliance.

**Methods:**

Prospective audit of patients seen in the breast clinic in Beaumont hospital over the week starting 8th February 2021. The medical charts were reviewed for documentation of chaperone presence. Doctors were surveyed using (SurveyMonkey) for causes of non-compliance. Interventions included a stamp in the medical notes for chaperone presence and details, an educational email with GMC and IMC guidelines, and posters put up in clinic rooms. The intervention was reassessed at 1-week and 6-week intervals.

**Results:**

In the assessment phase, 126 patients were recruited. A chaperone was present 100% of the time where a male doctor examined a female patient; however, chaperone presence was not documented in any of the medical charts (0/126). A survey was sent to 22 breast surgery doctors to explore causes of non-compliance. Response rate was 95%, 50% did not know documentation was necessary, and 25% forgot to document. One week after intervention, 64 patients were recruited. Chaperone documentation increased to 80% (51/64). Reassessment at six weeks included 120 patients, and chaperone documentation rate was 74% (89/120).

## Introduction

A chaperone is an impartial observer which should be a health professional. A chaperone should be sensitive and respect patient dignity and confidentiality, reassure patient if they show any signs of discomfort or distress and be familiar with the procedures involved in an intimate physical examination [1].

Intimate examinations can be uncomfortable and embarrassing for patients. According to the Irish Medical Council (IMC), a chaperone must be offered to all patients before intimate examinations, and the presence of a chaperone should be documented in the medical notes [2].

The general medical council (GMC) also recommends the presence of a chaperone during intimate examinations and that patients should be offered a chaperone irrespective of gender. GMC guidelines also recommend that a note should be made of the chaperone as well as their identity in the medical notes [1].

Both guidelines emphasize record keeping of the intimate examination and the presence of chaperone and their identity. Our aim was to assess and improve our breast clinic’s compliance with the GMC and IMC guidelines for documentation of chaperone use during breast examination.

## Methods

**Fig. 1:**
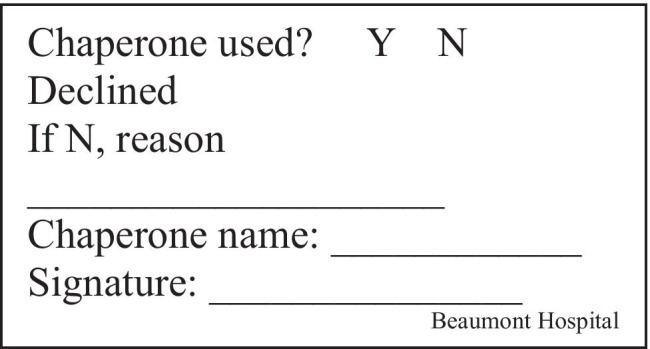
Chaperone stamp

**Fig. 2:**
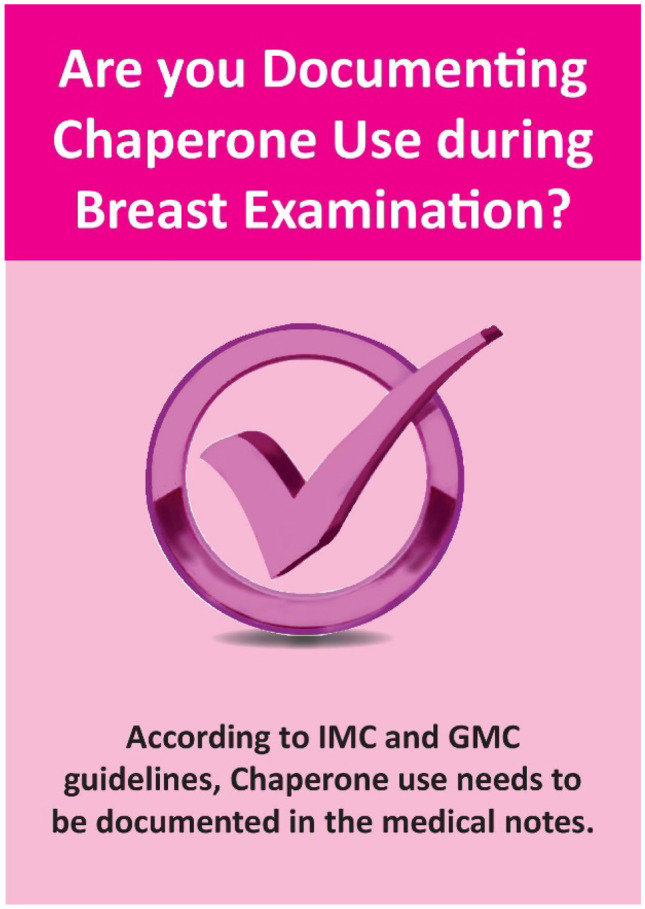
Poster for the breast clinic

This prospective audit recruited patients seen in the breast clinics in Beaumont hospital over the week starting 8th February 2021. Presence of chaperone with each patient is recorded, and the medical chart is reviewed for documentation of chaperone presence and name. Doctors were surveyed electronically using SurveyMonkey™ for causes of non-compliance. Descriptive analysis was done. Interventions included a stamp (Fig. 1) with chaperone name and details attached to patient notes as a reminder; an email reminder was sent to members of the breast team, and posters were put up in breast clinic rooms (Fig. 2). The intervention was reassessed at 1- and 6-week intervals.

## Results

A total of 126 patients were recruited before intervention. Of those 126 patients, 65 were seen by a male doctor (51.5%). Whilst a chaperone was present in all these consultations, this was not documented in any of their notes. Thirty-nine patients were seen by a female doctor (30.9%), and 22 were seen by both male and female doctors (17.4%).

A survey was conducted to explore possible causes for this non-compliance. Twenty one out of twenty-two doctors responded (95%). The causes identified were the following:

- 50% admitted that they did not know documentation was necessary.
- 19% stated that there is no reminder in the chart.
- 6% cited time and efficiency issues.
- 25% cited both time and efficiency issues as well as the absence of a reminder in the chart.

Reassessment of compliance with documenting chaperone presence was carried out 1 week after the introduction of the chaperone stamp. In total, 64 patients were recruited. Chaperone documentation was done in 80% (51/64) of those patients.

A further review to establish sustainability was carried out at 6 weeks after the introduction of our intervention. In total, 120 patients were recruited. Chaperone documentation was present in 74% (89/120) of those patients (Table 1).

**Table 1:** Documentation of chaperone presence before and after interventions

	Audit before intervention (*n* = 126)	Audit 1 week post-intervention (*n* = 64)	Audit 6 weeks post-intervention (*n* = 120)	Improvement (%)
Documentation of chaperone	0	51	89	80% at 1 week and 74% at 6 weeks

## Discussion

Medical Protection Society (MPS) supported the guidelines by the GMC on intimate examinations stating that the presence of chaperone adds a layer of protection for both patients and doctors despite the rarity of allegations of assault [3]. The presence of chaperone also acknowledges patient’s vulnerability and act to maintain their dignity at all times [3].

International guidelines have been developed to address intimate examinations. For instance, The American Medical Association Code of Medical Ethics recommends that all patients be offered an option to request the presence of a chaperone [4].

Sinha et al. [5] studied patient attitudes and preferences regarding the use of chaperone during breast examination. It found that 33% of patients (65 out of 200) preferred to have a chaperone while 65% (104 out of 200) felt they did not need one. A majority of patients in this study stated that the presence of chaperone made them feel at ease.

Despite that chaperone use was seen in 100% of male doctor examinations of female patients, documentation was non-existent in phase one of this audit. Forgetfulness and lack of education regarding this important guideline were the main reasons for poor compliance in our unit. International literature suggests this is not just a local issue. Rosenthal et al. [6] questionnaire of general practitioner found that 71% (884) of doctors did not record the identity of chaperone, and 66% (818) did not record the offer of chaperone.

The simple and cheap measures we implemented increased documentation from 0 to 80% at 1 week. The compliance rate was sustained at 74% in week six. Concentration fatigue, change of non-consultant hospital doctors (NCHDs) and forgetting to stamp medical notes by secretaries were among the reasons observed for this minor drop in compliance. Another assessment and audit will be carried out in a years’ time to establish these reasons objectively and improve the compliance rate further.

Overall, our figures compare satisfactorily with international standards. Rose et al. [7] carried out a single centre quality improvement project in a tertiary breast unit. Their interventions included discussion of chaperone guidelines alongside reminder posters and note stamp. They recorded a 69.95% documentation of chaperone in the 1st postintervention cycle and 74.86% in the 2nd postintervention cycle. This suggests that a proforma approach to chaperones is an effective means in ensuring adherence to best practice guidelines.

## Conclusion

Chaperone should be offered to all patients before performing intimate examinations. Doctors should be aware of the guidelines addressing intimate examination and preservation of patients’ dignity. Continuous education and medical notes prompting stamps are very helpful to maintain this necessary practice. This will lead to preserving patient dignity and improving overall patient satisfaction, and to more medico-legal protection for patients and doctors.
